# Fecal indicator organisms in northern oligotrophic rivers: An explorative study on *Escherichia coli* prevalence in a mountain region with intense tourism and reindeer herding

**DOI:** 10.1007/s10661-022-09865-1

**Published:** 2022-03-09

**Authors:** Sharon Maes, Monica Odlare, Anders Jonsson

**Affiliations:** Mid Sweden University, Faculty of Science, Technology and Media, Department of Ecotechnology and Sustainable Building Engineering, Akademigatan 1, 831 25 Östersund, Sweden

**Keywords:** Environmental monitoring, Fecal pollution, Principal component analysis, Sustainable water management

## Abstract

Increasing pollution levels in waters from remote mountain areas in northern Sweden have been observed. To support a sustainable water quality management, it is necessary to know which environmental and antrophogenic factors influence the water quality. The purpose of this study was to map the *Escherichia coli* prevalence in the catchment area of the upper part of a large northern Scandinavian river and investigate the controlling factors of microbial contamination. A total of 112 water samples were collected from various locations in the research area between July 2020 and December 2020. These samples were analyzed for microbial and chemical characteristics, and information about tourism and reindeer herding was compiled. Additionally, microbial and physicochemical water characteristics collected by Indalsälven Water Conservation Association (IWCA, 1993–2020) and Swedish Meteorological and Hydrological Institute (SMHI, 2004–2020) were analyzed. The results showed that *E. coli* enumerations ranged between 0 and 500 CFU/100 ml. There was generally no obvious relation between suspected point sources, e.g., sewage treatment plants at mountain stations, and *E. coli* levels at downstream sampling points. Principal component analysis showed that *E. coli* was correlated to coliforms, total heterotrophic count, river discharge, COD_Mn_ and river color. Since microbial analyses are time-consuming, expensive and difficult to perform in remote areas, it is important to find more easily extracted water parameters that can serve as a proxy for *E. coli*. In particular, river color and discharge are promising parameters that may serve as an early indication of bacterial outbreak and fecal contamination in mountain waters.

## Introduction

An important factor to maintain healthy ecosystems is water quality. Sufficiently high water quality is important to support the diversity of plants and wildlife, but is also critical to the health and welfare of human society. The naturally oligotrophic rivers in northern Sweden are generally characterized by a low pollution level (SLU, 2021; van Dijk et al., 1994), and the water quality, in remote (upper) parts of the catchment areas, may still meet national standards (Swedish Food Agengy, 2015) for drinking water (i.e., ≤ 10 CFU/100 mL *Escherichia coli* (*E. coli*) in water not intended for commercial or public use) without further treatment (Jonsson & Agerberg, 2015). The occurrence of such clean surface waters is a rather unique feature in the world and most likely depends on the few permanent settlements, remote location and difficult accessibility to these mountainous areas. According to the European Water Framework Directive (Directive 2000/60/EC, 2000), the member states have an obligation to ensure that the water quality of lakes and watercourses does not deteriorate based on current environmental status. Hence, Sweden has a special obligation to protect the good water quality in the large rivers of northern Scandinavia and thereby contribute to the UN Agenda 2030 Sustainable Development Goals, in particular target 6.6, “*protect and restore water-related ecosystems, including mountains, forests, wetlands, rivers, aquifers and lakes”* (United Nations, 2015).

Unfortunately, fecal indicator organisms such as *E. coli* are generally not included in national monitoring programs for Swedish rivers (SLU, 2021) nor are they included in the environmental quality standards that are currently used to assess ecological status of surface waters (Swedish Agency for Marine & Water Management, 2021). Nevertheless, an increasing trend in *E. coli* contamination has been observed in samples taken by the Indalsälven Water Conservation Association (IWCA) at locations in the most upstream catchment area of the river Indalsälven, one of the big rivers of central northern Sweden. Also, an increasing trend in samples that do not meet drinking water standards was observed over the years (IWCA, 2021). The change in microbial water quality will have a severe negative impact on the ecosystem, wild animals, visitors, inhabitants as well as indigenous people dependent on the land for their daily income, such as Sami herders. Moreover, the large number of inhabitants within the catchment area that are dependent of the river as a source of drinking water are exposed to serious risk through outbreak of waterborne disease such as the outbreak of *Cryptosporidium hominis* in Östersund in 2010 where almost half of the city’s population (27.000 out of 60.000) became ill (Lindberg et al., 2011).

To support a sustainable water quality management, it is necessary to know which factors have an influence on the water quality (Davis et al., 2014) and in this case especially on microbial contamination. Examples of possible influences on the water quality level in the upper parts of Indalsälven and similar northern Scandinavian rivers in the mountain regions are the increased pressure on scenic land sites close to rivers and lakes for permanent and holiday dwellings, increasing summer tourism, climate change and reindeer husbandry (Jonsson & Agerberg, 2015; Whitehead et al., 2009).

Currently, there is no information available on the microbiological contamination status of rivers and tributaries in the most upstream parts of the catchment area of Indalsälven. Consequently, is it not known what the source(s) of the elevated *E. coli* contamination levels could be. To investigate the current status of the microbiological water contamination in the most upstream catchment area of the river Indalsälven, *E. coli* levels were mapped. *E. coli* is, because of its presence in high numbers in the intestines of warm blooded animals (World Health Organization, 2020), used as indicator for water safety regarding fecal contamination and consequently recommended as most effective for predicting the presence of pathogens in water (Ashbolt et al., 2001; Directive 2009/54/EC on the Exploitation and Marketing of Natural Mineral Waters, 2009; Recreational Water Quality Criteria, 2012; Ishii & Sadowsky, 2008). Beside, data about water chemical and microbial characteristics collected by the IWCA and data about water flow rate by the Swedish Meteorological and Hydrological Institute (SMHI) were analyzed with the aim of acquiring important knowledge about the source and distribution patterns of fecal contamination. This information can serve as a base to develop a scheme for regular or continuous monitoring of the water quality in rivers in northern Sweden and for future water management strategies and mitigation measures in mountain areas worldwide that are increasingly being exploited for tourism.

The objectives of the present study are: (1) to do an initial mapping of the prevalence of *E. coli* in the research area and (2) identifying the controlling factors of this microbial contamination.

## Materials and methods

### Research area

**Fig. 1:**
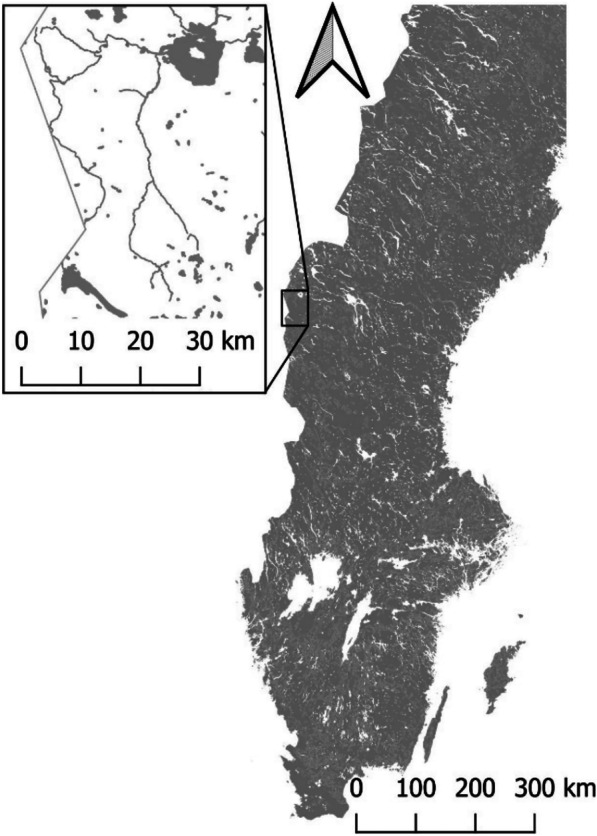
Sampling area consisting of the catchment area of the most upstream part of the river Indalsälven, Jämtland County, Sweden. The left part represents all waterways and lakes in the research area adjacent to the East Norwegian/West Swedish border. The right part represents a negative print of all waterways and lakes in Sweden

The focus area of this research is the catchment area of the most upstream part of the river Indalsälven, Jämtland County, Sweden, Europe (Fig. 1). This area is dominated by the river basins of two tributaries to Indalsälven, Enan and Handölan, surrounded by mountain ranges that raise above the tree limit to approximately 1700 m above sea level, which makes it a very remote area with few roads or permanent settlements. Tourism and reindeer herding by the Sami community are the main activities in this region.

The catchment areas of Enan and Handölan are 325km^2^ and 454km^2^, respectively. Most of this area consist of heath and forest land, bogs and wetland and bare mountains with thin soil. The ground mainly consists of moraine and thin soil on bare mountain, but also peat, coarse soil and glacial material. Enan has a yearly average water temperature of 3.1 °C and an average river flow rate of 10.7m^3^/s, while Handölan’s yearly average water temperature and river flow rate are 2.8 °C and 15.1m^3^/s (SMHI, 2021b). Other environmental characteristics of the research area are presented in Table 1. These data were collected at the nearest SMHI measuring location (i.e., Storlien) which is representative for the whole research area (SMHI, 2021a).

**Table 1:** General environmental characteristics of the research area in 2020 (SMHI, 2021a) including precipitation (mm and d), frost (d), sunshine (h), radiation (kWh/m^2^) and air temperature (°C)

**Month**	**Precipitation (mm)**	**Precipitation (d)**^**a**^	**Frost (d)**^**b**^	**Sunshine (h)**	**Radiation (kWh/m**^**2**^**)**	**Air temperature (°C)**
January	88	27	28	16	5.7	-0.6
February	71	23	29	68	25.5	-3.7
March	51	23	29	104	71.2	-2.8
April	71	23	26	108	116.1	0.1
May	56	23	24	172	179.6	2.3
June	59	8	1	373	202.9	14.2
July	78	24	3	125	117.8	9.4
August	47	16	3	183	111.4	11.6
September	72	22	4	109	60.0	7.1
October	57	23	10	56	24.8	3.2
November	64	22	16	43	8.6	1.1
December	16	19	30	4	2.9	-1.8
Yearly average	61	21	17	113	77.2	3.3

^a^Days with at least 0.1 mm precipitation

^b^Days with a minimum temperature below 0.0 °C

Based on geographical maps and locations of suspected pollution sources, sampling points were chosen considering aspects such as water flow rate, run-off areas, surrounding settlements, popular tourist locations and trails, reindeer fences, etc.

### Data collection in the research area

### Water sampling

In the period between July 2020 and December 2020, a total of 112 water samples were taken on 9 sampling moments to cover seasonal and temperature variations. To access the remote parts of the research area, it was necessary to use a terrain vehicle (Can-Am Outlander 6 × 6) equipped with a flatbed for transport of sampling equipment, electric cooler, extra battery, etc. Water was collected in sterilized plastic 500 mL bottles by completely submerging the bottles under the water surface with the opening facing upstream to avoid contamination by the sampler. Samples were taken by hand as far away from the shore as possible. After sampling, the water bottles were stored between 0 °C and 7 °C in an electric cooler. Time between sampling and microbiological analysis varied between 3h7min and 29h46min.

**Fig. 2:**
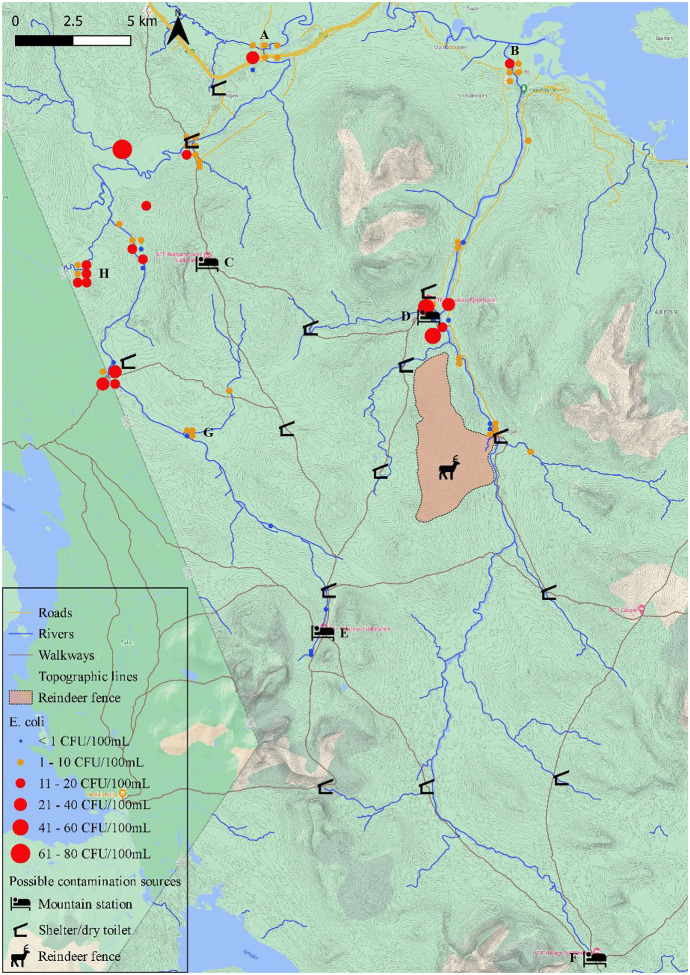
The river basins of Enan and Handölan with an indication of the rivers and tributaries, highways and roads, walkways, mountain stations, shelters with dry toilet, reindeer fence area and East Norwegian/West Swedish border. Results from *E. coli* enumerations from samples (n = 70) taken between July 2020 and December 2020 are indicated with dots, where the size of the dot is proportional to the *E. coli* level and the color indicates whether the water is pure (blue), acceptable (orange) or not suitable (red) for drinking according to national standards. A: sampling station for the IWCA control program at Enkroken, B: sampling location in Handöl village, C: Blåhammaren Mountain Station, D: Storulvån Mountain Station, E: Sylarna Mountain Station, F: Helags Mountain Station, G: first sampling across a river section (Klöftälven) and H: section sampling across a river section (Ranglan)

**Fig. 3:**
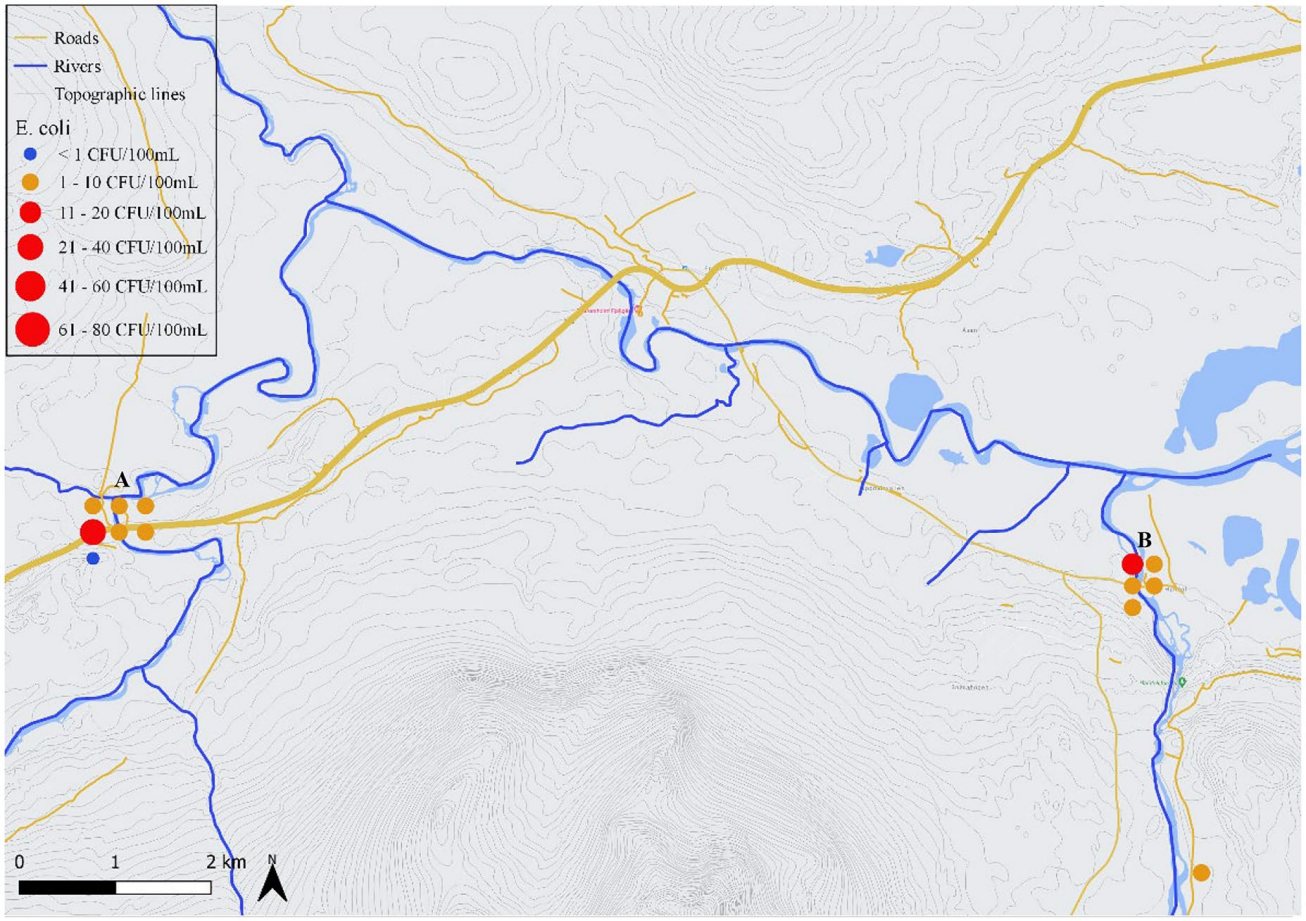
Sampling locations at Enkroken (A) and Handöl village (B) with an indication of the rivers and tributaries, highways and roads. Results from *E. coli* enumerations from samples (n = 13) taken between July 2020 and December 2020 are indicated with dots, where the size of the dot is proportional to the *E. coli* level and the color indicates whether the water is pure (blue), acceptable (orange) or not suitable (red) for drinking according to national standards

**Fig. 4:**
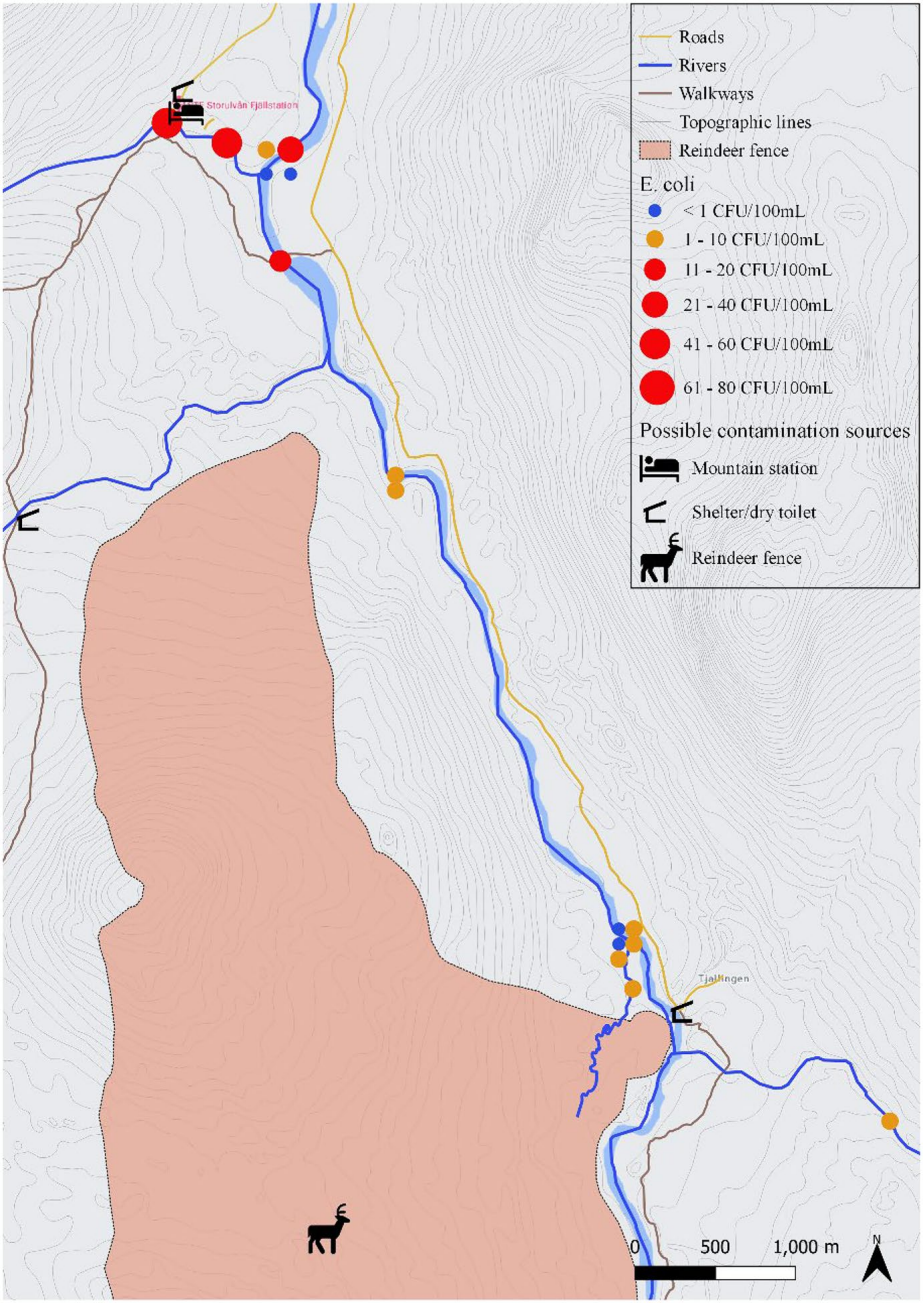
The river basin of Handölan around Tjallingen and Storulvån Mountain Station with an indication of the rivers and tributaries, roads, walkways, mountain station, shelters with dry toilet and reindeer fence area. Results from *E. coli* enumerations from samples (n = 16) taken between July 2020 and December 2020 are indicated with dots, where the size of the dot is proportional to the *E. coli* level and the color indicates whether the water is pure (blue), acceptable (orange) or not suitable (red) for drinking according to national standards

**Fig. 5:**
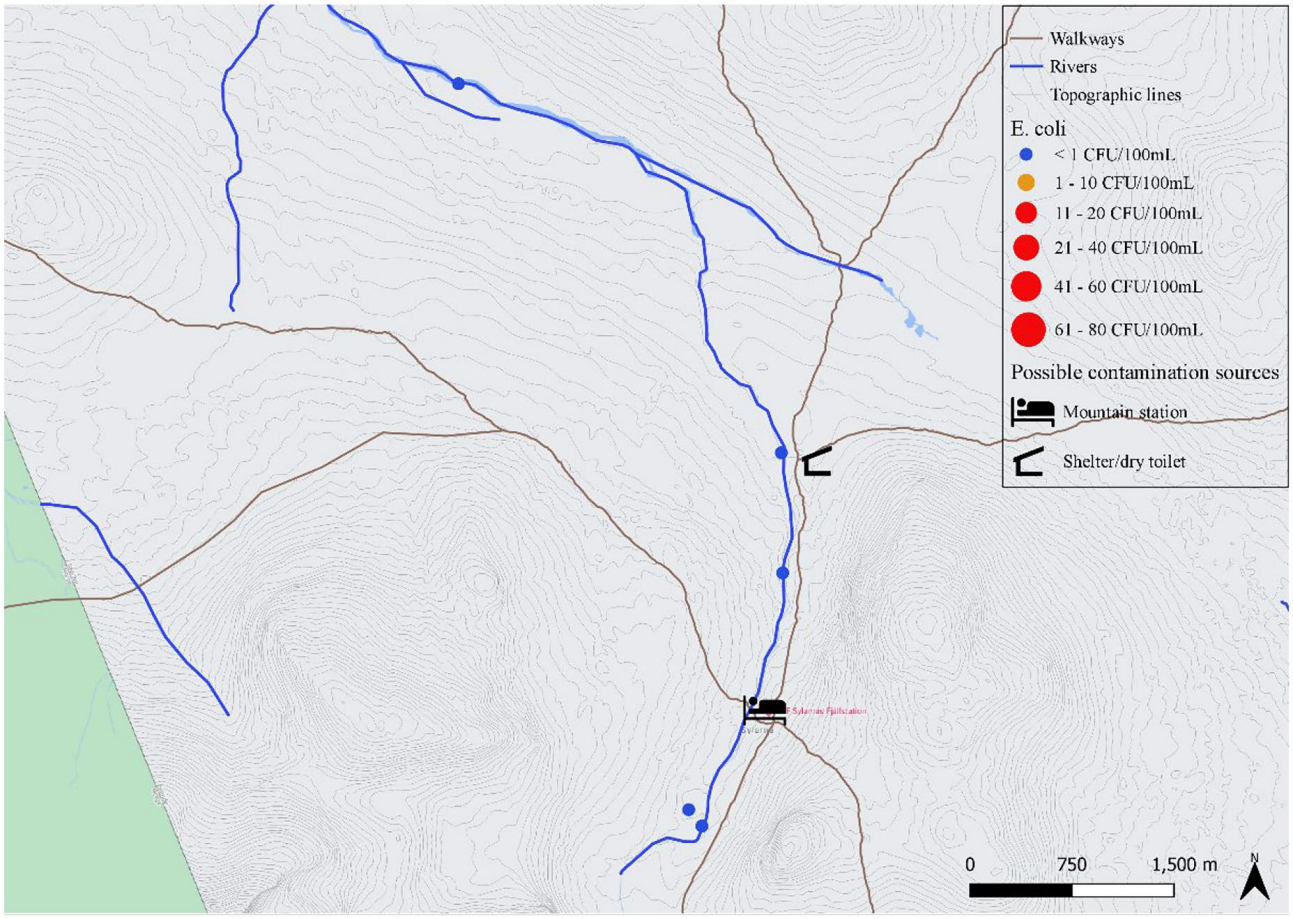
The river basin of Enan around Sylarna Mountain Station with an indication of the rivers and tributaries, walkways, mountain station, shelter with dry toilet and East Norwegian/West Swedish border. Results from *E. coli* enumerations from samples (n = 5) taken between July 2020 and December 2020 are indicated with dots, where the size of the dot is proportional to the *E. coli* level and the color indicates whether the water is pure (blue), acceptable (orange) or not suitable (red) for drinking according to national standards

**Fig. 6:**
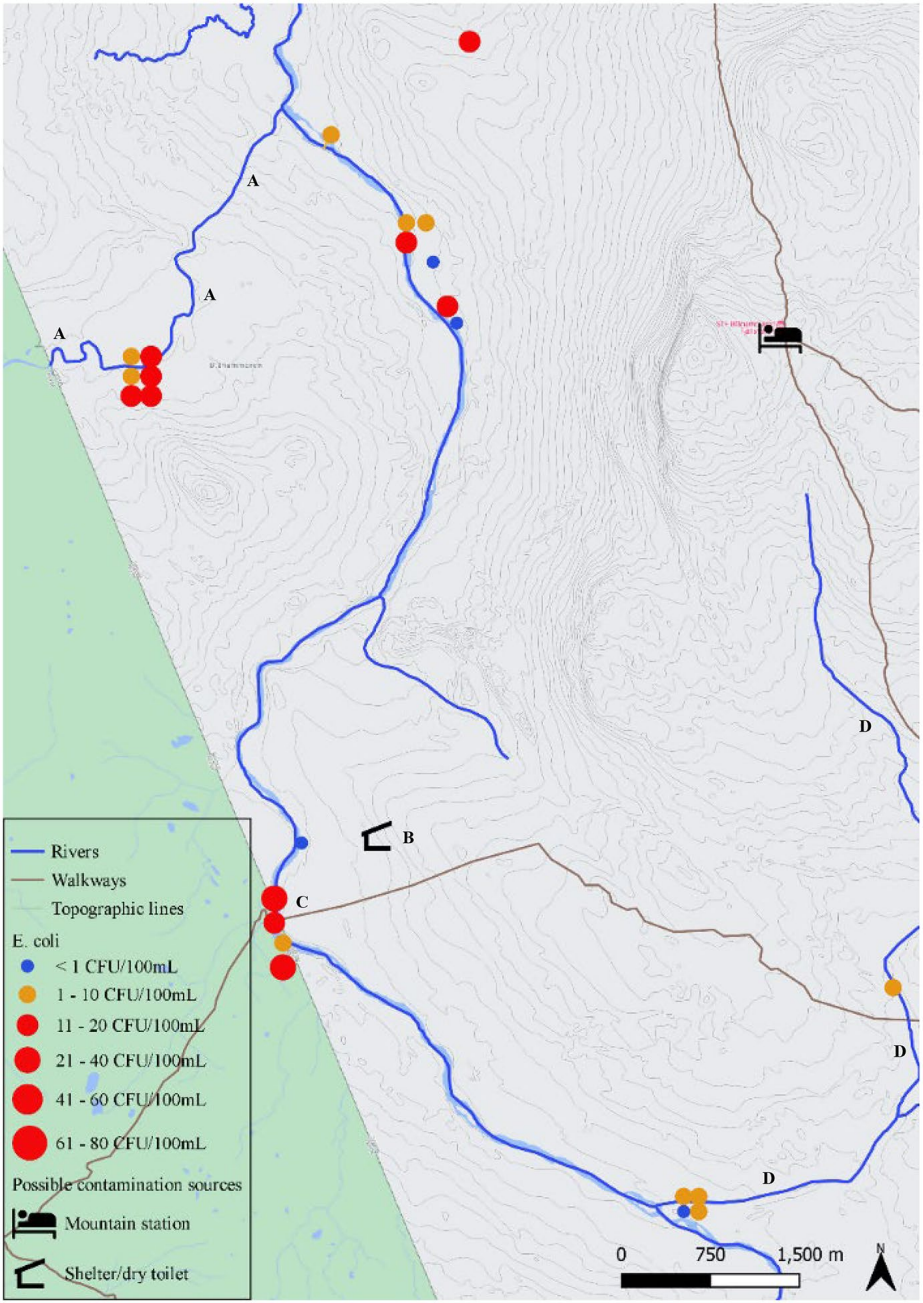
The river basin of Enan, West of Blåhammarens Mountain Station with an indication of the rivers and tributaries, walkways, mountain stations, shelters with dry toilet and East Norwegian/West Swedish border. Results from *E. coli* enumerations from samples (n = 24) taken between July 2020 and December 2020 are indicated with dots, where the size of the dot is proportional to the *E. coli* level and the color indicates whether the water is pure (blue), acceptable (orange) or not suitable (red) for drinking according to national standards. A: Tributary Ranglan, used for sampling across a river section, B: Stormklockan shelter, C: Södra Enbågen, D: Tributary Klöftälven, most downstream location was used for sampling across a river section

To map and discuss the microbiological contamination status of rivers and tributaries in the sampling area, a total of 70 unique water samples were used for *E. coli* analysis. Sixty-six of these samples were also analyzed for coliforms and 55 for total heterotrophic count (THC). The results of these *E. coli* enumerations were visualized on a map (Google Maps) using the Free and Open Source QGIS 3.16.1. The locations indicated on the maps (Figs. 2 to 6) might not be completely accurate but were chosen to give the best possible representation of the sampling spots. The exact locations together with other available information on the water samples taken in the sampling area are available in Table 2. The rest of the 112 samples were used for the evaluation of microbial and chemical distribution across a river section.

**Table 2:** Overview of 70 water samples taken in rivers and tributaries in the sampling area with information about location, sampling date, precipitation (mm), river flow rate (m^3^/s) and enumeration of *E. coli* (CFU/100 mL), coliforms (CFU/100 mL) and total heterotrophic count (THC, CFU/mL)

**Sampling location**	**Y-coordinate**	**X-coordinate**	**River Basin**	**Sampling date**	**Precipitation (mm)**	**River flow rate (m**^**3**^**/s)**	***E. coli* (CFU/100 mL)**	**Coliforms (CFU/100 mL)**	**THC (CFU/mL)**
Between Handöl village and Storulvån Mountain Station	63.19261	12.39036	Handölan	10-Jul-20	5,30	15,00	0	4	
Between Handöl village and Storulvån Mountain Station	63.19261	12.39036	Handölan	7-Jul-20	3,00	62,00	8		
Between Handöl village and Storulvån Mountain Station	63.19261	12.39036	Handölan	2-Oct-20	0,00	5,80	2	37	166
Handöl village	63.2605	12.43597	Handölan	10-Jul-20	5,20	16,00	1	2	
Handöl village	63.2605	12.43597	Handölan	7-Jul-20	3,00	65,00	11		
Handöl village	63.2605	12.43597	Handölan	2-Oct-20	0,00	6,40	8	15	77
Handöl village	63.2605	12.43597	Handölan	24-Nov-20	2,70	8,30	1	1	247
Handöl village	63.2605	12.43597	Handölan	14-Dec-20	2,20	1,50	1	3	462
Damaged bridge upstream Storulvån Mountain Station	63.161	12.37489	Handölan	7-Jul-20	3,00	50,00	14		
Storulvån Mountain Station, same side	63.16675	12.37428	Handölan	7-Jul-20	3,00	54,00	1		
Storulvån Mountain Station, same side	63.16675	12.37428	Handölan	2-Oct-20	0,00	5,10	25	36	184
Storulvån Mountain Station, opposite side	63.16631	12.37503	Handölan	10-Jul-20	5,40	13,00	0	4	
Storulvån Mountain Station, opposite side	63.16631	12.37503	Handölan	2-Oct-20	0,00	5,10	0	3	63
Downstream reindeer fence, East	63.1235	12.41833	Handölan	10-Jul-20	5,50	11,00	0	0	
Downstream reindeer fence, Mideast	63.12342	12.41808	Handölan	10-Jul-20	5,50	11,00	2	6	
Downstream reindeer fence, Midway	63.12336	12.41742	Handölan	10-Jul-20	5,50	11,00	0	2	
Downstream reindeer fence, Midwest	63.12322	12.41717	Handölan	10-Jul-20	5,50	11,00	1	2	
Downstream reindeer fence, West	63.12317	12.41694	Handölan	10-Jul-20	5,50	11,00	4	70	
Tributary reindeer fence	63.12042	12.41839	Handölan	10-Jul-20	4,70	0,14	4	68	
Tjallingån	63.11303	12.45011	Handölan	10-Jul-20	5,30	1,20	1	4	
Lillulvån	63.14906	12.38911	Handölan	10-Jul-20	5,40	10,80	4	6	
Lillulvån	63.14906	12.38911	Handölan	2-Oct-20	0,00	4,60	2	11	83
Storulvån, upstream sewage system	63.16869	12.36089	Handölan	2-Oct-20	0,00	0,38	49	49	106
Storulvån, downstream sewage system	63.16758	12.36828	Handölan	2-Oct-20	0,00	0,38	56	56	128
Beaver creek	63.23269	12.44803	Handölan	2-Oct-20			5	25	1280
Upstream Sylarna Mountain Station, same side^a^	63.03558	12.26375	Enan	27-Jul-20	4,40	0,92	0	0	61
Upstream Sylarna Mountain Station, opposite side^a^	63.03667	12.26183	Enan	27-Jul-20	4,40	0,92	0	0	38
Between Sylarna Mountain Station and Gamla Sylen^a^	63.05228	12.2755	Enan	27-Jul-20	4,40	0,92	0	0	51
Downstream Gamla Sylen^a^	63.06019	12.27531	Enan	27-Jul-20	4,40	0,92	0	10	44
Between Gamla Sylen and Sodra Enbågen	63.0845	12.22836	Enan	27-Jul-20	4,20	1,40	0	2	55
Upstream Sodra Enbågen	63.13986	12.09125	Enan	28-Jul-20	5,30	3,00	2	5	98
Upstream Sodra Enbågen	63.13986	12.09125	Enan	11-Aug-20	0,00	0,64	35	68	85
Downstream Sodra Enbågen, East	63.14319	12.09031	Enan	28-Jul-20	4,60	5,00	22	50	66
Downstream Sodra Enbågen, West	63.14322	12.08933	Enan	28-Jul-20	4,60	5,00	14	14	75
Ranglan, South	63.18322	12.06764	Enan	28-Jul-20	2,60	1,50	5	9	366
Ranglan, South^b^	63.18322	12.06764	Enan	11-Aug-20	0,21	0,22	14	202	361
Ranglan, South	63.18322	12.06764	Enan	24-Nov-20	6,40	1,20	4	4	775
Ranglan, Midsouth^b^	63.18322	12.06761	Enan	11-Aug-20	0,21	0,22	18	143	275
Ranglan, Midnorth^b^	63.18325	12.06761	Enan	11-Aug-20	0,21	0,22	18	157	378
Ranglan, North^b^	63.18328	12.06758	Enan	11-Aug-20	0,21	0,22	17	137	338
Bridge Enan	63.20064	12.09928	Enan	28-Jul-20	4,50	5,80	3	6	50
Upstream Sevedholm, South	63.23219	12.15817	Enan	28-Jul-20	4,00	7,80	10	10	151
Upstream Sevedholm, North	63.23161	12.15814	Enan	28-Jul-20	4,00	7,80	9	51	470
Downstream Sevedholm	63.2325	12.16169	Enan	28-Jul-20	3,70	9,40	4	16	275
Downstream Sevedholm	63.2325	12.16169	Enan	7-Aug-20	0,10	2,60	3	290	178
Downstream Sevedholm	63.2325	12.16169	Enan	11-Aug-20	0,10	1,70	13	79	321
Downstream Sevedholm	63.2325	12.16169	Enan	24-Nov-20	2,50	6,80	7	7	330
Enkroken	63.2645	12.22353	Enan	28-Jul-20	3,70	11,00	8	15	258
Enkroken	63.2645	12.22353	Enan	7-Aug-20	0,12	3,10	6	6	125
Enkroken	63.2645	12.22353	Enan	11-Aug-20	0,12	2,00	5	22	265
Enkroken^c^	63.2645	12.22353	Enan	17-Sep-20	1,60	16,00	27	313	1373
Enkroken	63.2645	12.22353	Enan	2-Oct-20	0,00	4,20	6	53	173
Enkroken	63.2645	12.22353	Enan	24-Nov-20	4,80	8,20	7	8	272
Enkroken	63.2645	12.22353	Enan	14-Dec-20	1,30	1,20	0	4	175
Creek West Blåhammaren Mountain Station, Collection	63.19386	12.11217	Enan	7-Aug-20			5	15	118
Creek West Blåhammaren Mountain Station, North	63.19306	12.114	Enan	7-Aug-20			2	23	146
Creek West Blåhammaren Mountain Station, Middle	63.19294	12.11422	Enan	7-Aug-20			11	13	134
Creek West Blåhammaren Mountain Station, South	63.19108	12.11628	Enan	7-Aug-20			0	19	130
Creek Northwest Blåhammaren Mountain Station	63.20764	12.12236	Enan	7-Aug-20			12	21	90
Creek North Blåhammaren Mountain Station	63.22478	12.16533	Enan	7-Aug-20			2	6	174
Tväråbäcken	63.22472	12.17014	Enan	7-Aug-20	0,21	0,10	5	9	100
Short creek North	63.18775	12.11867	Enan	7-Aug-20			11	36	171
Short creek South	63.1865	12.12022	Enan	7-Aug-20			0	5	131
Klöftälven upstream^a^	63.1365	12.19297	Enan	10-Aug-20	0,00	0,02	6	36	189
Tributary Stormklockan	63.14739	12.09436	Enan	11-Aug-20			0	16	20
Rekån	63.22931	12.10183	Enan	11-Aug-20	0,18	0,17	79	470	660
Klöftälven downstream, South^b^	63.12011	12.15933	Enan	10-Aug-20	0,04	0,16	1	7	127
Klöftälven downstream, Midsouth^b^	63.12022	12.15933	Enan	10-Aug-20	0,04	0,16	2	10	109
Klöftälven downstream, Midnorth^b^	63.12022	12.15928	Enan	10-Aug-20	0,04	0,16	2	15	128
Klöftälven downstream, North^b^	63.12025	12.15928	Enan	10-Aug-20	0,04	0,16	2	26	138

^a^The storage time of this sample exceeded 24 h

^b^Locations which were used for the evaluation of microbial and chemical distribution across a river section, results are the average of six consecutive water samples taken with 3 min time interval

^c^Results are the average of 3 consecutive samples taken with 3 min time interval

### Historical data collected at Enkroken

The sampling station at Enkroken (Figs. 2 and 3, X: 12.22353 Y: 63.26450) at the northern border of the research area collects the water from the catchment area of the river basin of Enan. This station serves as one out of 21 fixed stations for the IWCA’s control program for Indalsälven. Water samples have been taken here regularly since 1993 by IWCA. This generated data from 163 sampling occasions (until December 2020) of which 109 were analyzed for *E. coli* (IWCA, 2021). Twenty-seven of these samples were taken in February, 20 in March, 6 in April, 2 in June, 27 in August and 27 in October. These samples were also analyzed for other microbiological, chemical and physical parameters which are described in the sections “10” and “11.” A summary of these data can be consulted in Table 3.

### Evaluation of microbial and chemical distribution across a river section

The distribution of microbial and chemical parameters across river sections was evaluated at 2 sampling points (Figs. 2 and 6). These points were chosen based on their difference in river color according to visual evaluation. Six consecutive water samples were taken at four spots perpendicular to the river flow with 3 min time interval, covering a total of 15 min. The sampling in Klöftälven took place on August 10, 2020 and sampling in Ranglan took place on August 11, 2020.

### Collection of data on river flow rate and precipitation in the sampling area

Modeled data for precipitation and water flow rate at the corresponding sampling locations and days were retrieved from the SMHI website (SMHI, 2021b). The same information was collected for the location “Enkroken” since the start of the modeling in 2004. Calculations for sub-catchment areas were performed with the hydrological model S-HYPE. SMHI validated the model by the use of measured data at a number of stations along the river. The nearest point to Enkroken that was evaluated by the use of measured data on discharge and precipitation was Öster-Noren (X: 12.78855 Y: 63.43784) with a deviation of less than ± 10% of the measured value.

For each location, the total water flow was used. This represents the model-calculated water flow at the outlet of the chosen sub-catchment area including contributions from all possible sub-catchment areas upstream of the selected location.

### Knowledge about tourism and reindeer herding

According to the Swedish Tourist Association (Daniel Skog, Personal communication, October 3^rd^, 2020), the four mountain lodges in the area (Storulvån, Blåhammaren, Sylarna and Helags) were visited by 29.000 to 31.000 overnight guests during the summer season (June to October) of 2014–2020. The corresponding figures for the whole year were 36.000 to 44.000 overnight guests over the same time frame. The total number of people that visit the area is not known, but a survey made by the Department of Tourism and Geography at Mid Sweden University in the summer of 2013 indicates that 42% of the domestic overnight visitors and 29% of the foreign overnight visitors use the mountain lodges. The rest of the visitors stay in tents or cottages (Godtman-Kling, 2018). Based on these figures and including people coming to the area for day tours (walking and biking), a best guess of the total number of visitors that occupy the area during summer season is 100.000 or more.

The maximum allowed reindeer number owned by the Sami community (Handölsdalens Sameby) is 6000 in the winter heard (Annica Ideström, County Administrative Board of Jämtland, Personal communication, March 4^th^, 2021). The reindeer migrate over vast areas from their winter grazing grounds near the coast of the Baltic Sea, to the western mountains where they stay during spring and summer. In May, the female reindeer return to the research area to give birth to their calves high up in the mountain ranges. Approximately 3000 female reindeer and their calves are gathered in the fenced area at Tjallingen (Fig. 2) for one to two days in mid-July for marking of the calves (Jonas Kråik, Handölsdalens Sameby, Personal communication, May 10^th^, 2021).

### Microbiological analysis

Water samples collected in the sampling area were analyzed for *E. coli*, coliforms and THC. For enumerations of *E. coli* (based on SS-EN ISO 9308–1:2014), 10 mL and 100 mL of each water sample was filtered over a 0.45 µm filter (ReliaDisc^™^, Ahlstrom-Munksjö, 760,245). The filters were transferred to membrane fecal coliform agar (mFC, Difco^™^ mFC agar, BD Biosciences, 267,720) with 0.01% Rosolic acid (Difco^™^ Rosolic acid, BD Biosciences, 232,281) and incubated at 44 ± 0.5 °C for 22 ± 2 h. Suspicious *E. coli* colonies, which appear as blue on the mFC plate, were inoculated in a tube containing lactose tryptone lauryl sulfate broth (LTLSB, Oxoid, CM0921) and 4-methylumbelliferyl-β-D-glucuronide (MUG supplement, Oxoid, BR0071E) and incubated for 21 ± 3 h at 44 ± 0.5 °C for confirmation. Tubes that showed blue/green fluorescence under UV light were confirmed to contain *E. coli* strains. Confirmation was performed for all different (blue) morphologies which were visually observed on the mFC plates. Enumeration of coliforms was performed in a similar way but using m Endo Agar (Endo, Difco^™^ m Endo Agar LES, BD Biosciences, 273,620) as selective growth medium and incubation at 36 ± 1 °C for 22 ± 2 h. Colonies that were suspected to be coliforms appeared with a golden-green metallic sheen. Confirmation of these colonies was done based on the oxidase test (Bactident^®^ Oxidase, Merck Millipore, 1.13300.0001), at which coliforms generate a negative result. THC was performed by pour plating of 0.1 mL and 1 mL of each water sample using Water Plate Count Agar (wPCA, Oxoid, CM1012). Plates were incubated for 68 ± 4 h at 22 ± 1 °C after which all colonies were counted. The limit of quantification (LOQ) for enumerations of *E. coli* and coliforms was 1 CFU/100 mL and 1 CFU/mL for THC.

Microbiological analysis of the water samples collected at Enkroken by IWCA was performed by Hjortens Lab (Östersund). Enumeration of *E. coli* and coliforms was performed according to SS 028,167–2 MF and enumeration of THC according to (SS-EN) ISO 6222:1999.

The precision and accuracy of low count enumeration of *E. coli* in water was estimated by repeated analysis of a reference material for drinking water microbiology (Dw 2019:A, Livsmedelsverket, Swedish Food Agency) containing a certified value of 36 CFU/100 mL *E. coli*. A total of 22 aliquots from the reference material were analyzed at the laboratory by four different operators during the period September 2020–September 2021. The average value of the laboratory analyses was 36 CFU/100 mL with a standard deviation of 6.3 CFU/100 mL*.* The standard error of the mean was 1.4 CFU/100 mL, and the 95% confidence interval is 36 ± 2.8 CFU/100 mL *E. coli*.

### Physicochemical analysis

For a selection of the samples collected in the research area (n = 48), physical and chemical analysis was performed. These analyses were performed by Synlab Analytics and Services Sweden AB (Umeå). The turbidity was analyzed according to (SS-EN) ISO 7027–1: 2016, Chemical Oxygen Demand of Permanganate (COD_Mn_) according to SS 02 81 18 and Total Organic Carbon (TOC) according to (SS-EN) 1484:1997.

Chemical and physical analysis of the water samples collected at Enkroken by IWCA was also performed by Synlab Analytics and Services Sweden AB. Turbidity and COD_Mn_ were analyzed according to the previously mentioned methods and this was supplemented with the analysis of river color (according to (SS-EN) ISO 7887:2012 C mod 420 nm), total nitrogen (according to (SS-EN) ISO 11905–1:1997), total phosphorous (according to (SS-EN ISO) 15,681–2:2018), alkalinity (according to (SS-EN) ISO 9963–2), electrical conductivity (according to (SS-EN) 27,888–1) and pH (according to (SS-EN) ISO 10523:2012). Also, the water temperature was registered on the sampling location.

### Statistical analysis

For the evaluation of microbial and chemical distribution across a river section, one-way ANOVA at the 5% significance level followed by a post hoc test (Tukey’s HSD multiple comparison) was performed for each investigated parameter by using the software SPSS Statistics 27.

Multivariate statistics was performed through a Principal Component Analysis (PCA), which allows a visual presentation of relationships between samples and variables. The advantage of a PCA is that it can reveal patterns that may not be easily discovered when using classical statistics. In a PCA, a large dataset of possibly correlated variables is transformed into a new, smaller dataset. The transformation is performed by identifying directions, called principal components (PCs), where the maximum variation in the dataset can be found. The results from the PCA are presented as scores, describing variation in samples, and loadings, describing variations in variables. The confidence region in the PCA plots was based on Hotelling’s T2 test, which is a multivariate version of Student’s t test. The confidence limit was selected to be 95%. The PCA was performed using the software The Unscrambler X v. 10.5 (CAMO Software AS, Norway).

## Results

### Mapping of the prevalence of *E. coli* in the research area

### Water sampling

The results of *E. coli* enumerations are visualized on the map in Fig. 2 and were categorized in 3 groups, i.e., < 1 CFU/100 mL (blue), 1 to 10 CFU/100 mL (orange) and > 10 CFU/100 mL (red). The size of the symbol is also proportional to the *E. coli* level. The exact results of *E. coli* enumeration, other microbiological analysis and sampling details are available in Table 2.

The 2 locations that are closest to the connection between Indalsälven and the rivers Enan and Handölan are Enkroken and Handöl village, respectively (Fig. 3). Enkroken was sampled on 7 different sampling times, with *E. coli* values ranging from 0 to 25 CFU/100 mL and 8 CFU/mL on average. Values for *E. coli* were proportional to the number of coliforms and THC. At Handöl village, the average *E. coli* count was 4 CFU/mL ranging between 1 and 11 CFU/100 mL. The highest value at these 2 locations occurred when the flow rate was the highest (16 m^3^/s for Enkroken and 65 m^3^/s for Handöl village).

The most upstream location sampled in Handölan was Tjallingen (Fig. 4), where 5 samples were taken across the river next to the fenced area where reindeer were gathered for calve marking 3 days before sampling. *E. coli* enumeration ranged from 0 to 4 CFU/100 mL, the highest value being closest to a tributary coming from the fenced area which also contained 4 CFU/100 mL. The sample taken closest to the fenced area the morning after calve marking (approximately 4.5 km downstream) contained 14 CFU/100 mL. Unfortunately, on this occasion no samples were collected near the reindeer fence as intended. A broken bridge and high-water after heavy rainfall made it not possible to reach the fenced area that day. Further downstream Handölan, samples were taken around Storulvån Mountain Station. Differences in *E. coli* levels could be observed between the southeast (< 1 CFU/100 mL on both sampling occasions) and northwest (1 and 25 CFU/100 mL) bank of the river immediately downstream the tributary Stor-Ulvån. In this tributary, also two water samples were taken. One upstream and one downstream a creek coming from the direction of Storulvån’s sewage treatment plant was contaminated with 49 and 56 CFU/100 mL *E. coli*, respectively. Further downstream close to Handöl village (Fig. 3), a creek with active beaver traces contained 5 CFU/100 mL *E. coli* and a noticeably higher amount of THC (1280 CFU/mL).

Another river that was extensively sampled was Enan. The most upstream area where samples were taken was around Sylarna Mountain station (Fig. 5). No *E. coli* could be enumerated in the samples taken upstream Sylarna, and also in the downstream samples up to approximately 6 km, there was no *E. coli* found. This was always accompanied by no to low numbers of coliforms (0 to 10 CFU/100 mL) and low numbers of THC (38 to 61 CFU/mL).

Further downstream Enan (Fig. 6), several more samples were taken among others in the tributary Klöftälven. The upstream sample contained 6 CFU/100 mL *E. coli*, while the downstream location was used in the evaluation of microbiological and chemical distribution across a river section and contained 1 to 2 CFU/100 mL *E. coli* per sampling spot*.* Around a popular camping area along a trail connecting Sweden and Norway (i.e., Södra Enbågen), samples were taken up- and downstream a pedestrian bridge across Enan. Higher *E. coli* levels were found here, mostly exceeding the limit for drinking water even when the river flow rate was very low (e.g., 35 CFU/100 mL upstream the pedestrian bridge when the river flow rate was 0.64 m^3^/s, while the average river flow rate from 1981 until 2019 was 4.6 m^3^/s at this location). No *E. coli* were found in a small creek (not visible on the map) coming from the direction of a shelter along the trail (i.e., Stormklockan). The second location where an evaluation of microbiological and chemical distribution across a river section was performed, was tributary Ranglan. *E. coli* enumeration varied from 14 to 18 CFU/100 mL per sampling spot and THC was noticeably higher in this tributary. Several creeks West of Blåhammaren Mountain Station were sampled, providing mixed results ranging between < 1 to 11 CFU/100 mL *E. coli* and moderate THC numbers. The same was true for 3 creeks to the north of Blåhammaren.

The water sample that contained the most *E. coli* originated from tributary Rekån (Fig. 2). This sample carried 79 CFU/100 mL *E. coli* and also the number of coliforms was much higher compared to other samples (470 CFU/100 mL) while the river flow rate was low (0.17 m^3^/s). Samples taken around a very popular rest area with shelter (i.e., Sevedholm) provided *E. coli* enumerations ranging between 3 and 13 CFU/100 mL and slightly higher than moderate numbers for THC.

### Historical data collected at Enkroken

**Fig. 7:**
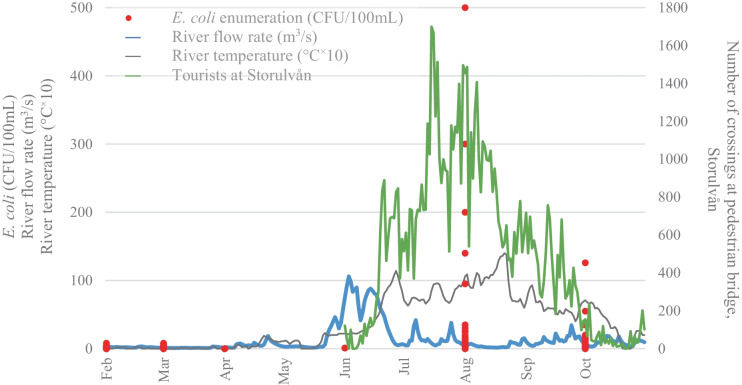
*E. coli* enumeration (CFU/100 mL, red dots) in water samples taken at Enkroken per month over a period of 1993 to 2020, supplemented with modeled data about the river flow rate (m^3^/s, blue curve) and river temperature (°C^×^10, grey curve) at Enkroken in 2020 and the number of crossings over the pedestrian bridge at Storulvån Mountain Station (green curve) from June until October 2020

The IWCA sampling station at Enkroken (IWCA, 2021) is representative for the upper parts of river Indalsälven and clearly shows the oligotrophic properties of this cold and very clear water (Table 3). The river is in its upper parts characterized by ultra-oligotrophy with total-phosphorous frequently far below 6 µg/L. The river also has very low alkalinity and turbidity, and low concentrations of organic matter and color. What has been puzzling for many years is the frequently elevated levels of fecal indicator organisms such as *E. coli*. The level of *E. coli* at Enkroken is frequently even higher than at further downstream, and more inhabited, parts of the river. *E. coli* enumeration at station Enkroken from 1993 to 2020 ranged between 0 and 500 CFU/100 mL (Fig. 7). The highest values are observed for the samples taken in the months August and October. It should be noted in this context that no samples from January, May, July, September, November and December were included in the monitoring program. The average/**median** *E. coli* numbers over the years were 1/**1** CFU/100 mL in February (n = 27), 2/**1** CFU/100 mL in March (n = 20), 0/**0** CFU/100 mL in April (n = 6), 1/**1** CFU/100 mL in June (n = 2), 56/**15** CFU/100 mL in August (n = 27) and 16/**6** CFU/100 mL in October (n = 27). The national standards for drinking water, i.e., ≤ 10 CFU/100 mL *E. coli* in water not intended for commercial or public use, are often exceeded in the months August and October.

**Table 3:** Water physicochemical and microbial characteristics of samples collected between 1993 and 2020 at Enkroken (IWCA, 2021). For each analyzed parameter, average, standard deviation, median, minimum, maximum and sample size are given

**Water parameter**	**Average**	** ± **	**Stdev**	**Median**	**Min**	**-**	**Max**	**n**
Temperature (°C)	2.7	±	4.1	0.5	-0.5	**-**	15.4	141
Alkalinity (mekv/L)	0.278	±	0.130	0.250	0.047	**-**	0.590	163
COD_Mn_ (mg/L)	2.6	±	1.5	2.4	0.0	**-**	7.6	163
Color (mg/L Pt)	20	±	12	15	0	**-**	60	161
Conductivity (mS/m)	4.1	±	1.5	3.8	1.3	**-**	7.5	163
pH	7.3	±	0.3	7.3	6.3	**-**	8.5	163
Total-P (µg/L)	3.9	±	2.9	3.0	0.0	**-**	17.0	163
Turbidity (FNU)	0.46	±	0.38	0.34	0.17	**-**	3.50	162
Total heterotrophs (CFU/mL)	612	±	1101	300	49	**-**	8690	110
Coliform bacteria (CFU/100 mL)	60	±	193	13	0	**-**	1720	110
*E. coli* (CFU 100/mL)	19	±	61	2	0	**-**	500	109

### Evaluation of microbial and chemical distribution across a river section

The distribution of microbiological and chemical parameters was evaluated in the tributaries Klöftälven and Ranglan (Table 4). The average *E. coli* enumeration on 4 locations across Klöftälven ranged between 1 and 2 CFU/100 mL, while the Ranglan samples contained on average between 14 and 18 CFU/100 mL *E. coli*. The enumeration of *E. coli* for the different sampling times ranged between 0 and 3 CFU/100 mL in Klöftälven and between 14 and 19 CFU/100 mL in Ranglan. A one-way ANOVA was conducted to compare the effect of sampling spot and sampling time on the enumeration of *E. coli*, coliforms and THC and on turbidity, COD_Mn_ and TOC. There was no significant effect of sampling spot on *E. coli* enumerations in Klöftälven and Ranglan. However, there was a significant difference between the sampling spots in Klöftälven for coliforms (p < 0.001) and in Ranglan for THC (p = 0.012). For the chemical parameters analyzed in these 2 river crossings, only a significant difference for COD_Mn_ between the locations in Klöftälven (p < 0.001) was established. There was never a significant difference observed between the sampling times for all investigated parameters in both tributaries.

**Table 4:** Microbiological (*E. coli* (CFU/100 mL), coliforms (CFU/100 mL) and THC (CFU/mL)) and chemical (turbidity (FNU), COD_Mn_ (mg/L) and TOC (mg/L)) analysis at 4 locations (A-D) across the tributaries Klöftälven and Ranglan and on 6 times (T1-T6) with 3 min time interval. Values represent the mean ± standard deviation per parameter per location (n = 6) or time (n = 4) and statistically significant differences (Tukey p = 0.05) per parameter are indicated by different letters for the sampling spots or times

		**Location**													**Time**																	
		**A**			**B**			**C**			**D**				**T1**			**T2**			**T3**			**T4**			**T5**			**T6**		
**Klöftälven**																																
	*E. coli*	2	±	4	2	±	4	2	±	1	1	±	1		3	±	5	2	±	0	0	±	0	1	±	1	3	±	5	0	±	1
	Coliforms	26^a^	±	6	15^b^	±	10	10^b^	±	3	7^b^	±	3		13	±	11	13	±	10	20	±	15	16	±	11	12	±	8	13	±	2
	THC	138	±	24	128	±	22	109	±	13	127	±	38		120	±	14	109	±	24	144	±	20	154	±	37	113	±	16	113	±	16
	Turbidity	0.14	±	0.02	0.18	±	0.01	0.13	±	0.01	0.20	±	0.17		0.20	±	0.09	0.13	±	0.01	0.25	±	0.2	0.13	±	0.03	0.14	±	0.02	0.12	±	0.02
	COD_Mn_	1.8^a^	±	0.1	1.8^a^	±	0.1	1.8^a^	±	0.1	1.6^b^	±	0.1		1.8	±	0.2	1.8	±	0.1	1.7	±	0.1	1.7	±	0.0	1.7	±	0.1	1.7	±	0.1
	TOC	1.6	±	0.1	1.6	±	0.1	1.6	±	0.1	1.6	±	0.1		1.6	±	0.0	1.7	±	0.1	1.5	±	0.1	1.7	±	0.1	1.6	±	0.1	1.5	±	0.1
**Ranglan**																																
	*E. coli*	17	±	6	18	±	3	18	±	6	14	±	6		18	±	7	14	±	6	16	±	3	18	±	3	19	±	5	18	±	8
	Coliforms	137	±	73	157	±	27	143	±	27	202	±	70		125	±	45	165	±	61	188	±	92	190	±	54	168	±	38	123	±	19
	THC	339^a,b^	±	50	378^b^	±	38	275^a^	±	51	361^b^	±	61		372	±	31	282	±	59	333	±	43	354	±	72	321	±	81	367	±	60
	Turbidity	0.49	±	0.02	0.49	±	0.03	0.49	±	0.02	0.48	±	0.01		0.48	±	0.01	0.49	±	0.02	0.49	±	0.02	0.49	±	0.02	0.48	±	0.01	0.50	±	0.03
	COD_Mn_	3.6	±	0.1	3.5	±	0.1	3.5	±	0.1	3.5	±	0.1		3.5	±	0.1	3.5	±	0.1	3.6	±	0.1	3.6	±	0.1	3.5	±	0.2	3.4	±	0.2
	TOC	2.7	±	0.1	2.7	±	0.1	2.7	±	0.1	2.8	±	0.2		2.8	±	0.1	2.8	±	0.1	2.6	±	0.2	2.7	±	0.1	2.8	±	0.1	2.7	±	0.2

### Identification of factors controlling microbial contamination in the research area

### Suspected sources within the research area

The considered suspected sources of fecal contamination within the research area are indicated in Figs. 2 to 6. The first group of suspected point sources for human fecal pollution are the four mountain stations in the area, i.e., Storulvån, Sylarna, Helags and Blåhammaren. Statistics from the Swedish Tourist Association (STF) show that the total number of overnight guests is approximately 30.000 per summer season and 36.000 to 44.000 per year (Personal communication, STF, Daniel Skog, March 9^th^, 2021). There are also an unknown number of visitors to the area that either camp by themselves or make day trips by walking or biking along the many trails in the area.

The sewage systems at Storulvån and Blåhammaren are similar and consist of a mechanical and chemical treatment of the sewage followed by infiltration. At Sylarna, sewage is biologically and chemically treated before infiltration and at Helags only a biological treatment is performed before infiltration. Other suspected point sources of human fecal pollution are the 14 shelters with dry toilets that are under surveillance of the county administration of Jämtland. Human feces is collected in a dug pit under the dry toilet where the fecal remains are composted. Diffuse fecal pollution from human sources is also possible from popular resting/camping places along the trails where there are no dry toilets. A suspected point source of animal fecal pollution is the reindeer fence at Tjallingen where approximately 3000 reindeer are fenced for marking of the calves. This fencing is only carried out for one to two days each summer in July. During the rest of the summer season, the reindeer are spread out over vast areas and contribute only as diffuse sources of animal fecal pollution. During our excursions in the area, beaver activity was noticed in the tributaries to both Handölan and Enan. Beavers are mammals that spend a large time of their life in the water and are considered a suspected source of animal fecal pollution to the water.

### Correlation between river characteristics and *E. coli*

Figure 7 shows, together with the results of *E. coli* enumeration in samples taken at station Enkroken from 1993 to 2020 (IWCA), data for the river flow rate (m^3^/s) and river temperature (°C × 10) at Enkroken in 2020 and the number of crossings over the pedestrian bridge at Storulvån Mountain Station from June until October 2020 (Data retrieved from the County administration board, personal communication, Lansstyrelsen, Wictoria Wadman, January 8^th^, 2021). The number of visitors crossing the pedestrian bridge at Storulvån during the summer of 2020 reaches a peak in July and August, whereas the river flow rate reaches a maximum in June.

During microbial sampling in mountain areas, the source of pollution can sometimes be obscured by uncontrollable factors such as temperature, water flow rate or precipitation. This is demonstrated by the significant correlation between data from *E. coli* enumeration and river water flow rate at Enkroken over a period of 2004 to 2020. The highest *E. coli* enumerations always occurred when the river flow rate was elevated (higher than 10m^3^/s, which was only the case for 10% of the measurements).

**Fig. 8:**
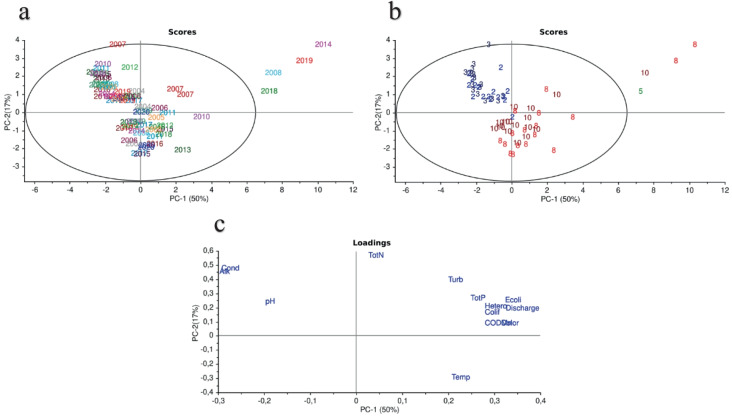
PCA plots of principal component 1 (PC-1) versus principal component 2 (PC-2) for chemical, physical and microbiological data at Enkroken. (a) Score plot with water samples labeled as (2004–2020) for the corresponding sampling year, (b) Score plot with water samples labeled as (2–10) for the corresponding sampling month and (c) loading plot with water variables (Alk: alkalinity, Cond: conductivity, pH, TotN: total nitrogen, Turb: turbidity, TotP: total phosphorous, *E. coli*, Hetero: total heterotrophic count, Colif: coliforms, Discharge, CODMn, Color and Temp: temperature)

Figure 8 shows scores and loadings plots of a principal component analysis of the physicochemical and microbiological variables investigated at Enkroken. The scores in a and b have been labeled based on the corresponding sampling year and month, respectively. The explanatory level was 50% for PC1 and 17% for PC2, which is high for data that are generated in a natural sampling setting such as in this study. The scores in plot a show that the different sampling years were not clearly clustered in different group, suggesting that there was no obvious difference between the sampling years when all variables were considered at the same time. However, scores in plot b show that the different sampling months were clustered together in 2 distinct groups, i.e., the cluster with samples taken in February/March (2/3) and the cluster with samples taken in August/October (8/10). This suggests that there is a difference in water samples taken between different sampling months when all variables were considered at the same time. Four samples can be found outside the confidence limit of 95%. It concerns the 4 samples with the highest river flow rate. The loading plot (c) shows that the variables *E. coli*, coliforms, THC, discharge (i.e. river flow rate), COD_Mn_ and river color are located close to each other, which indicates they are strongly correlated. This group of variables is to some extent related to total phosphorous and turbidity.

## Discussion

### Mapping of the prevalence of *E. coli* in the research area

This explorative study in the most upstream catchment area of a northern oligotrophic river reveals a complex pattern with respect to *E. coli* prevalence. *E. coli* numbers exceeding the national drinking water standards for private consumption were frequently registered, but we also found low or no *E. coli* at other sampling points.

On the other hand, interesting knowledge was gathered concerning sampling in remote mountain areas. The large distances, few roads and the mountainous terrain place great demand on any attempt to establish a monitoring program for *E. coli* in such a remote area. The use of helicopter is an expensive venture that most projects cannot afford. Even with an off-road vehicle, time and number of samples that could be collected per sampling effort were limiting factors. Off-road vehicles may cause severe damage to sensitive soil in the many wetland areas and should therefore be used cautiously. The time lapse between sampling and arrival at the laboratory is a crucial factor to take into consideration in the planning of sampling campaigns for bacterial enumerations. The Swedish standard for water surveys/sampling for microbiological analysis (SS-EN ISO 19458:2006) recommends analysis within 12 h of sampling but 18 h is also still accepted. Experiments were carried out in the laboratory to investigate the effect of storage on *E. coli* enumeration and the results indicated an accelerated inactivation of *E. coli* after 24 h of storage at 4 to 8ºC (unpublished data). Hence, it was necessary to carefully evaluate the landscape and the suspected sources of fecal contamination to select appropriate sampling points that could be sampled and analyzed within 24 h. This time limit was kept for most samples taken in the area but 5 out of 112 samples had to be stored between 24 and 30 h because of the transport distance. These samples were collected in the upper Enan area (4 samples around Sylarna Mountain Station, < 1 CFU/100 mL *E. coli*) and the upstream sample in Klöftälven near Blåhammaren Mountain Station (1 sample, 6 CFU/100 mL *E. coli*). Hence, it cannot be precluded that the bacterial counts in these 5 samples may have been underestimated.

The cross-sectional distribution experiments carried out in the two tributaries to Enan (Klöftälven and Ranglan) reveal some interesting aspects about water composition in relation to variability in time and space. While *E. coli* was evenly distributed in both space (across the tributary) and time (covering 15 min) in both rivers, this was not the case for coliforms (Klöftälven) or THC (Ranglan). These results suggest that *E. coli* comes from a point further upstream compared to coliforms in Klöftälven and THC in Ranglan. Hence, the source of *E. coli* could be sufficiently far away from the sampling location to allow for complete mixing across the river section. In their investigation of the Norwalk river (Connecticut), Crosby et al. (2019) also found minimal fluctuations of the bacterial concentrations at the sub-hourly and hourly timescales at each sampling point. The author’s conclusion that sampling from either side of the river results in comparable detection likelihoods was, however, not fully supported by our study. On the contrary, sampling both banks of the river may be a valuable tool to assess the influence of a suspected point source such as the creek coming from the fenced area for reindeer marking or even more so at the entry of tributary Stor-Ulvån into Handölan at Storulvån Mountain Station. The sample taken at the nearest point downstream the connection between Handölan and the tributary Stor-Ulvån (carrying 49 to 56 CFU/100 mL from the suspected point source at the sewage treatment plant) contained 25 CFU/100 mL at the tributary side while the sample taken on the opposite side contained < 1 CFU/100 mL. In these cases, the suspected source was close by (less than 10 m) and mixing of the bacteria across the river had not taken place at the sampling points.

Coliforms that are abundant in ageing feces and manure, have multiple non-point sources from, e.g., reindeer droppings in the terrain closer to the sampling point. While the water in Klöftälven was extremely clear with low concentrations of organic matter and color, the water in Ranglan was apparently more colored with higher content of organic matter. The origin of this organic matter is decaying plant material from the vast peatland in the sub-catchment area of Ranglan. The heterotrophic organisms, which are naturally occurring degrading organisms in the peatland, are brought to the sampling point at Ranglan from non-point sources in the surrounding peatland. The spatial distribution of the heterotrophs may therefore reflect differences in the physical properties of the riverbanks on both sides of the river, e.g. minor creeks entering organic rich water to the river causing uneven distribution of the heterotrophs at the local scale.

### Identification of factors controlling microbial contamination in the research area

As previously stated, results of *E. coli* enumeration in the research area varied a lot. Low or no *E. coli* were found at sampling points where fecal pollution could be expected, e.g. close to the sewage treatment plant at Sylarna Mountain Station, whereas high *E. coli* numbers were found on locations without an obvious and/or near source. Hence, there was not always an obvious connection between suspected point sources, e.g., the sewage treatment plants at mountain stations, and *E. coli* levels at downstream sampling points. The variability of *E. coli* is influenced by several factors that regulate both discharge of *E. coli* to the recipient water (e.g. fecal load to the sewage system, precipitation and surface runoff) and the inactivation rate of the bacteria (e.g. temperature, abiotic and biotic factors) (Blaustein et al., 2013). In cold and clear waters, such as northern oligotrophic rivers, the inactivation rate of *E. coli* is expected to be low, with inactivation rate constants k_20_ = 0,145 day^−1^ and k_20_ = 0,230 day^−1^ for pristine water and Indalsälven, respectively (Blaustein et al., 2013; Jonsson & Agerberg, 2015), leading to possibilities for considerable transport distances for the bacteria in the rivers depending on stream velocity. Hence, sources of fecal pollution could be rather far upstream in the system in relation to the sampling point, which means that multiple sources of fecal pollution might influence the bacterial numbers even at sampling points near suspected point sources.

If the infiltration step in the sewage treatment from the mountain stations works properly, *E. coli* and potential pathogens are expected to be inactivated before the percolating water from the infiltration bed reaches the recipient water (Stevik et al., 2004). This biological purification process in the infiltration bed is of course negatively influenced by the low temperatures that, even frequently during the summer months, prevail in the sampling area. Hence, lower *E. coli* inactivation rates than usual are expected during the infiltration of treated sewage water from the plants at the mountain stations. On the other hand, the retention time in the infiltration bed is crucial. If the retention time is too low, the risk of *E. coli* being washed out to the recipient increases. The combination of lower *E. coli* inactivation rates and insufficient retention leads to an increased risk of leakage of fecal pollution and *E. coli* to the recipient water. Precipitation and number of tourists at the mountain stations are key drivers that influence the retention time in the infiltration bed. Studies from the Norwalk river in Connecticut, USA (Crosby et al., 2019) and Göta älv river, Southwestern Sweden (Tornevi et al., 2014) both illustrate a significant positive relationship between the levels of fecal pollution indicators (such as *E. coli*) and precipitation. Studies carried out in rivers, creeks and estuaries in western Australia (Masters et al., 2011) and at beach sites in the Oslo fjord, Norway (Eregno et al., 2018) also showed strong links between precipitation and elevated levels of *E. coli* in the water. The combination of wet weather with heavy precipitation (when tourists tend to make more use of indoor facilities) and high visitor numbers in the area may explain the peak loads of *E. coli* that were found occasionally (especially in August which is the top tourist season) at Enkroken in the IWCA monitoring program since 1993. This matter will be further discussed below. It is in this context noteworthy that Crosby et al. (2019), although over 1900 samples were collected and analyzed between 2007 to 2017, infrequently found elevated bacterial counts during weekly and monthly monitoring. On finer spatial scales, the authors found that less than 25% of the original source concentration was detected at 10 m downstream and less than 10% by 1 km downstream a point source, suggesting that both high density of sampling sites and high frequency sampling may be needed to detect potential sources. It is not likely that such ambitious monitoring programs, how well-motivated they may be, can be realized in remote areas, for practical and economic reasons. In our field study, it is likely that the *E. coli* found at some sampling points are influenced by both point and diffuse sources and that the enumerated *E. coli* may be of both human and animal origin. Environmental DNA (eDNA) samples taken by the Swedish Natural History Museum (Källman, 2019) in river Enan at three occasions (August 14, October 16 and December 04, 2018; n = 7) showed that all samples were dominated by human DNA but also in decreasing order by other mammals such as beaver, reindeer, dog, pig and mice. It should be noted in this context that the proportion of people to reindeer in the area during summer period is greater than 10 and probably somewhere between 20 and 30, see section “9.”

The visitor’s peak in August (Fig. 7) seems to correlate with the frequently high counts of *E. coli* at Enkroken in the August samples. Together with the high number of reindeer present in the research area in the summer, it is clear that a higher deposit of fecal contamination in summer can be expected compared to the other (colder) seasons. In October, when the reindeer are still present but not so many visitors remain in the area, bacterial counts at Enkroken are still notably high although not as high as in August. The reason for the remaining high *E. coli* counts after the visitor's peak in August may be multifactorial. A combination of different variables can cause integrated effects that are not easily understood by analyzing one variable at a time. In these cases, multivariate statistical methods are useful when evaluating how several data may interact with each other. Methods such as PCA can reveal structures and statistical patterns in field observations and chemical and microbiological water data that may be missed if only classical statistical methods are used. The reason principal components are used is to deal with correlated predictors (multicollinearity) and to visualize data in a two-dimensional space. PCA is a statistical procedure to convert observations of possibly correlated features to principal components such that are uncorrelated with each other, are linear combinations of original variables, and help in capturing maximum information in the dataset. Here, the goal was to find variables that are easy and cost-effective to sample and analyze and correlate to *E. coli* measurements. Finding such proxies of microbial pollution might significantly increase the chances to make reliable predictions about the water quality status. From the PCA loading plot it appears that the variables river color, COD_Mn_ and discharge are closely related to the presence of *E. coli* as well as the other microbial measurements. Both river color and COD_Mn_ are measures of dissolved humic substances and the results from the PCA suggests that both variables potentially could be used as a proxy for the level of *E. coli* in areas where more precise microbiological measurements are difficult to implement. Since river color is relatively easy to measure on a continuous basis by measuring the absorbance at 420 nm, this may be an easy-to-use alternative to more elaborate methods based on portable fluorescence sensing, combined with advanced modeling methods to compensate readings for environmental interferences and false positives (Offenbaume et al., 2020). Also, river discharge is a cheap and easy to measure parameter which can be monitored on a continuous basis. As previously discussed, both high-frequency and high-density monitoring is most likely needed to be able to more precisely track the suspected sources of fecal pollution. Our research suggests that the change of color or discharge in clear and oligotrophic rivers may be a highly sensitive proxy for fecal contamination and that strategically placed monitoring stations for continuous monitoring of river color and discharge may provide an early-warning system for upstream outbreaks of bacteria and fecal contamination. We therefore suggest that such a monitoring system based on color and discharge should be validated in clear and oligotrophic rivers to assess their potential as an early-warning system for *E. coli* outbreaks in remote areas. This would, however, most likely not be enough to accurately track the sources of fecal pollution mainly because of difficulty to establish sufficiently high-density monitoring programs. One definite step forward would be to be able to separate the sources of *E. coli* from human and animal origin. This may be achieved by strategic sampling of eDNA (at “hotspots”) and microbial source tracking of sampled *E. coli* strains originating from water samples that were collected in this research area.

## Conclusion

In this research, we found that *E. coli* prevalence in the catchment area of the most upstream part of the river Indalsälven (Jämtland County, Sweden) was highly variable in time and space, indicating increased levels at some locations. Despite the challenges a remote area such as this one poses, our results covered a large variation in sampling year, month, temperature, UV exposure, etc., providing representative results for the area. In addition to regular microbiological analysis, the measurement of river color and flow rate was suggested as interesting variables for continuous measurement as a proxy for fecal contamination in the river system including the possibility for an early warning system for hazardous microbial outbreaks.

In order to maintain the high water quality in this oligotrophic river system, the identification of fecal contamination source(s) is crucial. This is not only important to limit or prevent the discharge of fecal pollution into the river system but also to estimate the danger that this contamination can pose to human health. However, based solely on structured water sampling, it is still very difficult to pinpoint the sources of fecal pollution. Therefore, in future research the focus will be on the identification of sources of fecal pollution in this area. This will be investigated by the combined analysis of eDNA and microbial source tracking of the collected *E. coli* strains originating from water samples taken in this research area.

## Data Availability

All data generated or analyzed during this study are included in this published article or available from the authors.

## Code availability

Microsoft Office Professional Plus 2019, SPSS Statistics 27 and Unscrambler X v. 10.5 were used for analysis.
